# Supplementary material for: Hot spot prediction in protein-protein interactions by an ensemble system
Source: BMC Syst Biol. 2018 Dec 31;12(Suppl 9):132. doi: 10.1186/s12918-018-0665-8 (PMC6311905; doi:10.1186/s12918-018-0665-8)
Supplement: Supplementary file 2 — All relevant MATLAB code. A simple Matlab implement of our predictor. (ZIP 19,742 kb) [file 12918_2018_665_MOESM2_ESM.zip › Additional file 2/Read ASA/ASA website/BID/1NFIF.htm]

 NetSurfP - Protein Surface Accessibility and Secondary Structure Predictions

  

|  |  |  |
| --- | --- | --- |
|  |  | NetSurfP - Protein Surface Accessibility and Secondary Structure Predictions  Technical University of Denmark |

  


---

```
# For publication of results, please cite:
# A generic method for assignment of reliability scores applied to solvent accessibility predictions.
# Bent Petersen, Thomas Nordahl Petersen, Pernille Andersen, Morten Nielsen and Claus Lundegaard
# BMC Structural Biology 2009, 9:51 doi:10.1186/1472-6807-9-51
#
# Column 1: Class assignment - B for buried or E for Exposed - Threshold: 25% exposure, but not based on RSA
# Column 2: Amino acid
# Column 3: Sequence name
# Column 4: Amino acid number
# Column 5: Relative Surface Accessibility - RSA
# Column 6: Absolute Surface Accessibility
# Column 7: Z-fit score for RSA prediction
# Column 8: Probability for Alpha-Helix
# Column 9: Probability for Beta-strand
# Column 10: Probability for Coil
E L  1NFIF                  1    0.568 104.019  -1.502   0.003   0.003   0.994
E T  1NFIF                  2    0.302  41.846  -0.815   0.018   0.047   0.935
E E  1NFIF                  3    0.661 115.442  -0.587   0.115   0.016   0.868
E D  1NFIF                  4    0.580  83.621  -0.317   0.115   0.016   0.868
E G  1NFIF                  5    0.327  25.774  -0.779   0.109   0.005   0.886
E D  1NFIF                  6    0.300  43.172   0.887   0.181   0.016   0.803
B S  1NFIF                  7    0.097  11.310   0.252   0.354   0.048   0.598
B F  1NFIF                  8    0.034   6.864   0.673   0.831   0.044   0.125
B L  1NFIF                  9    0.023   4.266   0.905   0.911   0.033   0.057
B H  1NFIF                 10    0.089  16.225   0.562   0.938   0.007   0.055
B L  1NFIF                 11    0.098  17.944   1.314   0.975   0.003   0.022
B A  1NFIF                 12    0.018   1.951   1.038   0.975   0.003   0.022
B I  1NFIF                 13    0.023   4.347   0.343   0.938   0.007   0.055
E I  1NFIF                 14    0.362  66.951   0.673   0.879   0.010   0.111
E H  1NFIF                 15    0.412  74.870   0.340   0.430   0.016   0.555
E E  1NFIF                 16    0.488  85.166  -1.571   0.016   0.005   0.979
B E  1NFIF                 17    0.246  42.959  -0.392   0.016   0.005   0.979
E K  1NFIF                 18    0.552 113.588  -0.133   0.184   0.043   0.773
E A  1NFIF                 19    0.505  55.629  -0.546   0.257   0.016   0.727
B L  1NFIF                 20    0.259  47.405   0.381   0.430   0.016   0.555
B T  1NFIF                 21    0.094  12.968   0.034   0.858   0.002   0.139
B M  1NFIF                 22    0.194  38.839   0.573   0.923   0.002   0.076
E E  1NFIF                 23    0.261  45.614   0.731   0.970   0.001   0.030
B V  1NFIF                 24    0.033   5.103   0.575   0.975   0.003   0.022
B I  1NFIF                 25    0.022   4.089   1.409   0.975   0.003   0.022
E R  1NFIF                 26    0.303  69.364   1.765   0.975   0.003   0.022
B Q  1NFIF                 27    0.093  16.646   1.369   0.975   0.003   0.022
B V  1NFIF                 28    0.027   4.196   1.033   0.975   0.003   0.022
B K  1NFIF                 29    0.099  20.385   0.278   0.938   0.007   0.055
E G  1NFIF                 30    0.470  37.020   0.468   0.782   0.003   0.216
E D  1NFIF                 31    0.293  42.164   1.046   0.321   0.003   0.675
E L  1NFIF                 32    0.404  74.064  -1.358   0.053   0.005   0.942
B A  1NFIF                 33    0.045   4.904  -0.288   0.018   0.019   0.964
E F  1NFIF                 34    0.468  94.028   0.647   0.018   0.088   0.893
B L  1NFIF                 35    0.165  30.175   0.974   0.019   0.141   0.840
E N  1NFIF                 36    0.381  55.778   0.354   0.020   0.205   0.775
E F  1NFIF                 37    0.414  83.150   0.387   0.056   0.142   0.802
E Q  1NFIF                 38    0.572 102.123   0.776   0.056   0.142   0.802
E N  1NFIF                 39    0.322  47.082  -0.534   0.053   0.043   0.903
E N  1NFIF                 40    0.647  94.692  -1.439   0.058   0.017   0.925
E L  1NFIF                 41    0.565 103.488  -0.593   0.018   0.019   0.964
E Q  1NFIF                 42    0.355  63.439  -0.327   0.016   0.005   0.979
B Q  1NFIF                 43    0.217  38.685   0.792   0.115   0.016   0.868
B T  1NFIF                 44    0.052   7.143   0.425   0.278   0.093   0.628
B P  1NFIF                 45    0.027   3.775   0.904   0.779   0.100   0.120
B L  1NFIF                 46    0.020   3.625   1.126   0.911   0.033   0.057
B H  1NFIF                 47    0.092  16.644   0.598   0.938   0.007   0.055
B L  1NFIF                 48    0.096  17.541   1.022   0.975   0.003   0.022
B A  1NFIF                 49    0.019   2.094   0.797   0.938   0.007   0.055
B V  1NFIF                 50    0.028   4.319  -0.083   0.938   0.007   0.055
E I  1NFIF                 51    0.434  80.309   0.648   0.802   0.014   0.185
E T  1NFIF                 52    0.452  62.665   0.725   0.321   0.003   0.675
E N  1NFIF                 53    0.494  72.336  -0.794   0.016   0.005   0.979
B Q  1NFIF                 54    0.141  25.254   0.363   0.109   0.005   0.886
E P  1NFIF                 55    0.330  46.841   1.254   0.938   0.007   0.055
E E  1NFIF                 56    0.360  62.892   1.092   0.950   0.028   0.022
B I  1NFIF                 57    0.037   6.771   1.161   0.950   0.028   0.022
B A  1NFIF                 58    0.027   2.953   1.191   0.950   0.028   0.022
E E  1NFIF                 59    0.364  63.556   1.855   0.950   0.028   0.022
B A  1NFIF                 60    0.118  13.037   1.580   0.950   0.028   0.022
B L  1NFIF                 61    0.038   6.994   0.959   0.938   0.007   0.055
B L  1NFIF                 62    0.108  19.811   0.596   0.938   0.007   0.055
E G  1NFIF                 63    0.467  36.784   0.677   0.802   0.014   0.185
E A  1NFIF                 64    0.282  31.131   1.157   0.430   0.016   0.555
E G  1NFIF                 65    0.385  30.323  -1.407   0.115   0.016   0.868
B C  1NFIF                 66    0.059   8.227   0.023   0.018   0.019   0.964
E D  1NFIF                 67    0.472  67.943   0.627   0.018   0.088   0.893
B P  1NFIF                 68    0.214  30.338   1.101   0.056   0.142   0.802
E E  1NFIF                 69    0.466  81.480   0.201   0.056   0.142   0.802
E L  1NFIF                 70    0.422  77.195   0.858   0.056   0.142   0.802
E R  1NFIF                 71    0.534 122.217   0.538   0.113   0.087   0.800
E D  1NFIF                 72    0.267  38.417  -0.297   0.053   0.043   0.903
E F  1NFIF                 73    0.591 118.654  -0.940   0.058   0.017   0.925
E R  1NFIF                 74    0.527 120.683  -0.310   0.018   0.019   0.964
E G  1NFIF                 75    0.359  28.222  -0.434   0.016   0.005   0.979
E N  1NFIF                 76    0.248  36.278   0.816   0.115   0.016   0.868
B T  1NFIF                 77    0.059   8.239   0.433   0.278   0.093   0.628
B P  1NFIF                 78    0.027   3.789   0.888   0.751   0.050   0.199
B L  1NFIF                 79    0.023   4.248   0.945   0.911   0.033   0.057
B H  1NFIF                 80    0.097  17.662   0.641   0.938   0.007   0.055
B L  1NFIF                 81    0.087  15.985   1.307   0.975   0.003   0.022
B A  1NFIF                 82    0.019   2.149   0.870   0.975   0.003   0.022
B C  1NFIF                 83    0.029   4.114  -0.023   0.938   0.007   0.055
E E  1NFIF                 84    0.418  73.094   0.600   0.802   0.014   0.185
E Q  1NFIF                 85    0.456  81.477   0.521   0.321   0.003   0.675
E G  1NFIF                 86    0.512  40.255  -0.982   0.016   0.005   0.979
B C  1NFIF                 87    0.131  18.435   0.418   0.109   0.005   0.886
E L  1NFIF                 88    0.345  63.115   1.134   0.879   0.010   0.111
E A  1NFIF                 89    0.302  33.247   0.930   0.911   0.033   0.057
B S  1NFIF                 90    0.033   3.844   0.746   0.918   0.063   0.019
B V  1NFIF                 91    0.028   4.304   1.073   0.950   0.028   0.022
E G  1NFIF                 92    0.381  30.016   1.696   0.950   0.028   0.022
B V  1NFIF                 93    0.107  16.461   1.587   0.950   0.028   0.022
B L  1NFIF                 94    0.030   5.438   0.952   0.950   0.028   0.022
B T  1NFIF                 95    0.093  12.830   0.599   0.879   0.010   0.111
E Q  1NFIF                 96    0.349  62.331   0.638   0.802   0.014   0.185
B S  1NFIF                 97    0.227  26.651   1.506   0.430   0.016   0.555
B C  1NFIF                 98    0.216  30.326  -0.962   0.115   0.016   0.868
B T  1NFIF                 99    0.046   6.408  -0.574   0.018   0.019   0.964
E T  1NFIF                100    0.398  55.133   0.361   0.018   0.047   0.935
E P  1NFIF                101    0.373  52.915   0.337   0.053   0.043   0.903
E H  1NFIF                102    0.606 110.322  -0.817   0.058   0.017   0.925
E L  1NFIF                103    0.502  91.880  -0.263   0.058   0.017   0.925
E H  1NFIF                104    0.580 105.502  -0.783   0.053   0.043   0.903
E S  1NFIF                105    0.376  44.079  -0.356   0.056   0.142   0.802
E I  1NFIF                106    0.453  83.823   0.256   0.066   0.296   0.638
B L  1NFIF                107    0.110  20.178  -0.172   0.069   0.386   0.545
E K  1NFIF                108    0.301  61.977  -0.607   0.074   0.484   0.442
E A  1NFIF                109    0.369  40.675  -0.329   0.069   0.386   0.545
E T  1NFIF                110    0.459  63.705   0.127   0.066   0.296   0.638
E N  1NFIF                111    0.298  43.642  -0.541   0.056   0.142   0.802
E Y  1NFIF                112    0.586 125.143  -1.369   0.053   0.043   0.903
E N  1NFIF                113    0.532  77.841  -0.673   0.018   0.019   0.964
E G  1NFIF                114    0.313  24.641  -1.029   0.018   0.019   0.964
E H  1NFIF                115    0.232  42.274   0.879   0.113   0.043   0.844
B T  1NFIF                116    0.048   6.602  -0.079   0.307   0.165   0.527
B C  1NFIF                117    0.023   3.271   1.170   0.649   0.163   0.188
B L  1NFIF                118    0.021   3.790   1.155   0.779   0.100   0.120
B H  1NFIF                119    0.090  16.298   0.792   0.911   0.033   0.057
B L  1NFIF                120    0.088  16.113   1.323   0.950   0.028   0.022
B A  1NFIF                121    0.018   1.951   0.866   0.950   0.028   0.022
B S  1NFIF                122    0.028   3.305  -0.170   0.911   0.033   0.057
E I  1NFIF                123    0.424  78.384   0.331   0.802   0.014   0.185
E H  1NFIF                124    0.459  83.401   0.239   0.257   0.016   0.727
E G  1NFIF                125    0.522  41.066  -1.141   0.016   0.005   0.979
B Y  1NFIF                126    0.194  41.522   0.411   0.053   0.005   0.942
E L  1NFIF                127    0.307  56.248   1.105   0.831   0.044   0.125
E G  1NFIF                128    0.318  25.066   0.909   0.870   0.077   0.053
B I  1NFIF                129    0.032   5.902   0.916   0.918   0.063   0.019
B V  1NFIF                130    0.025   3.766   1.244   0.918   0.063   0.019
E E  1NFIF                131    0.345  60.254   1.625   0.950   0.028   0.022
B L  1NFIF                132    0.122  22.393   1.604   0.911   0.033   0.057
B L  1NFIF                133    0.033   6.042   0.932   0.911   0.033   0.057
B V  1NFIF                134    0.101  15.554   0.188   0.879   0.010   0.111
E S  1NFIF                135    0.448  52.470   0.442   0.717   0.014   0.269
E L  1NFIF                136    0.279  51.067   1.231   0.339   0.016   0.645
E G  1NFIF                137    0.409  32.212  -1.388   0.058   0.017   0.925
B A  1NFIF                138    0.060   6.590   0.235   0.018   0.019   0.964
E D  1NFIF                139    0.472  68.030   0.545   0.018   0.088   0.893
B V  1NFIF                140    0.187  28.742   0.904   0.056   0.142   0.802
E N  1NFIF                141    0.377  55.178   0.213   0.056   0.142   0.802
E A  1NFIF                142    0.447  49.226   0.708   0.113   0.087   0.800
E Q  1NFIF                143    0.500  89.300   0.399   0.113   0.087   0.800
E E  1NFIF                144    0.396  69.164  -0.367   0.113   0.043   0.844
E P  1NFIF                145    0.561  79.592  -0.583   0.058   0.017   0.925
E C  1NFIF                146    0.506  70.972  -0.702   0.058   0.017   0.925
E N  1NFIF                147    0.489  71.516  -0.210   0.018   0.019   0.964
B G  1NFIF                148    0.159  12.498  -1.465   0.018   0.019   0.964
B R  1NFIF                149    0.222  50.861   0.816   0.184   0.043   0.773
B T  1NFIF                150    0.046   6.325   0.413   0.278   0.093   0.628
B A  1NFIF                151    0.026   2.887   0.884   0.831   0.044   0.125
B L  1NFIF                152    0.020   3.625   1.165   0.938   0.007   0.055
B H  1NFIF                153    0.079  14.443   0.624   0.975   0.003   0.022
B L  1NFIF                154    0.097  17.834   1.209   0.975   0.003   0.022
B A  1NFIF                155    0.021   2.336   0.718   0.938   0.007   0.055
B V  1NFIF                156    0.025   3.889   0.314   0.938   0.007   0.055
E D  1NFIF                157    0.398  57.352   0.372   0.802   0.014   0.185
E L  1NFIF                158    0.480  87.943   0.281   0.321   0.003   0.675
E Q  1NFIF                159    0.535  95.569  -0.935   0.016   0.005   0.979
B N  1NFIF                160    0.155  22.707   0.258   0.109   0.005   0.886
E P  1NFIF                161    0.363  51.467   1.280   0.938   0.007   0.055
E D  1NFIF                162    0.339  48.835   1.117   0.938   0.007   0.055
B L  1NFIF                163    0.032   5.896   0.989   0.950   0.028   0.022
B V  1NFIF                164    0.027   4.165   1.083   0.975   0.003   0.022
E S  1NFIF                165    0.381  44.606   1.690   0.975   0.003   0.022
B L  1NFIF                166    0.117  21.441   1.650   0.975   0.003   0.022
B L  1NFIF                167    0.035   6.463   0.923   0.938   0.007   0.055
B L  1NFIF                168    0.110  20.178   0.479   0.938   0.007   0.055
E K  1NFIF                169    0.442  90.817   0.488   0.802   0.014   0.185
E C  1NFIF                170    0.270  37.880   1.317   0.430   0.016   0.555
E G  1NFIF                171    0.430  33.865  -1.372   0.058   0.017   0.925
B A  1NFIF                172    0.051   5.598   0.083   0.018   0.019   0.964
E D  1NFIF                173    0.445  64.067   0.390   0.052   0.084   0.864
B V  1NFIF                174    0.224  34.413   1.158   0.056   0.142   0.802
E N  1NFIF                175    0.465  68.017   0.200   0.056   0.142   0.802
E R  1NFIF                176    0.403  92.264   1.023   0.056   0.142   0.802
E V  1NFIF                177    0.554  85.227   0.706   0.118   0.150   0.732
E T  1NFIF                178    0.299  41.499   0.029   0.053   0.043   0.903
E Y  1NFIF                179    0.630 134.716  -0.991   0.058   0.017   0.925
E Q  1NFIF                180    0.547  97.641  -0.080   0.018   0.019   0.964
E G  1NFIF                181    0.370  29.095  -0.322   0.016   0.005   0.979
E Y  1NFIF                182    0.254  54.344   0.959   0.115   0.016   0.868
B S  1NFIF                183    0.083   9.728   0.386   0.268   0.043   0.689
B P  1NFIF                184    0.031   4.413   0.644   0.831   0.044   0.125
B Y  1NFIF                185    0.026   5.578   0.812   0.879   0.010   0.111
B Q  1NFIF                186    0.138  24.736   0.797   0.938   0.007   0.055
B L  1NFIF                187    0.098  18.017   1.416   0.975   0.003   0.022
B T  1NFIF                188    0.018   2.427   0.733   0.938   0.007   0.055
B W  1NFIF                189    0.065  15.729  -0.281   0.938   0.007   0.055
E G  1NFIF                190    0.519  40.806   0.293   0.802   0.014   0.185
E R  1NFIF                191    0.496 113.538   0.474   0.246   0.004   0.750
E P  1NFIF                192    0.590  83.764  -1.096   0.016   0.005   0.979
B S  1NFIF                193    0.175  20.522   0.336   0.053   0.005   0.942
E T  1NFIF                194    0.402  55.771   1.517   0.879   0.010   0.111
E R  1NFIF                195    0.368  84.295   1.251   0.879   0.010   0.111
B I  1NFIF                196    0.030   5.606   1.033   0.950   0.028   0.022
B Q  1NFIF                197    0.036   6.483   0.852   0.938   0.007   0.055
E Q  1NFIF                198    0.445  79.423   1.843   0.975   0.003   0.022
B Q  1NFIF                199    0.122  21.789   1.710   0.975   0.003   0.022
B L  1NFIF                200    0.034   6.225   0.853   0.975   0.003   0.022
B G  1NFIF                201    0.145  11.427   0.600   0.938   0.007   0.055
E Q  1NFIF                202    0.478  85.407   0.830   0.879   0.010   0.111
E L  1NFIF                203    0.271  49.565   1.774   0.502   0.002   0.495
E T  1NFIF                204    0.344  47.754  -1.184   0.176   0.004   0.820
B L  1NFIF                205    0.067  12.176  -0.055   0.115   0.016   0.868
E E  1NFIF                206    0.568  99.230   1.185   0.268   0.043   0.689
E N  1NFIF                207    0.382  55.939   1.162   0.354   0.048   0.598
E L  1NFIF                208    0.390  71.372   0.067   0.339   0.016   0.645
E Q  1NFIF                209    0.652 116.429   0.667   0.339   0.016   0.645
E M  1NFIF                210    0.559 111.756   1.053   0.257   0.016   0.727
E L  1NFIF                211    0.502  92.008   0.383   0.115   0.016   0.868
E P  1NFIF                212    0.645  91.483  -0.262   0.058   0.017   0.925
E E  1NFIF                213    0.917 160.200   0.990   0.003   0.003   0.994

Explain the output.  Go back.
```

---
